# The serotonin 5-HT3 receptor: a novel neurodevelopmental target

**DOI:** 10.3389/fncel.2013.00076

**Published:** 2013-05-27

**Authors:** Mareen Engel, Marten P. Smidt, Johannes A. van Hooft

**Affiliations:** ^1^Center for NeuroScience, Swammerdam Institute for Life Sciences, University of AmsterdamAmsterdam, Netherlands; ^2^Max Planck Institute of PsychiatryMunich, Germany

**Keywords:** serotonin, 5-HT_3_ receptor, development, interneurons, neuroblasts

## Abstract

Serotonin (5-hydroxytryptamine, 5-HT), next to being an important neurotransmitter, recently gained attention as a key-regulator of pre- and postnatal development in the mammalian central nervous system (CNS). Several receptors for 5-HT are expressed in the developing brain including a ligand-gated ion channel, the 5-HT_3_ receptor. Over the past years, evidence has been accumulating that 5-HT_3_ receptors are involved in the regulation of neurodevelopment by serotonin. Here, we review the spatial and temporal expression patterns of 5-HT_3_ receptors in the pre- and early postnatal rodent brain and its functional implications. First, 5-HT_3_ receptors are expressed on GABAergic interneurons in neocortex and limbic structures derived from the caudal ganglionic eminence. Mature inhibitory GABAergic interneurons fine-tune neuronal excitability and thus are crucial for the physiological function of the brain. Second, 5-HT_3_ receptors are expressed on specific glutamatergic neurons, Cajal–Retzius cells in the cortex and granule cells in the cerebellum, where they regulate morphology, positioning, and connectivity of the local microcircuitry. Taken together, the 5-HT_3_ receptor emerges as a potential key-regulator of network formation and function in the CNS, which could have a major impact on our understanding of neurodevelopmental disorders in which 5-HT plays a role.

## INTRODUCTION

In addition to its role as a classical neurotransmitter, it is now well established that serotonin (5-hydroxytryptamine, 5-HT) plays a pivotal role in the development of the mammalian central nervous system (CNS). 5-HT is one of the first neurotransmitters to appear during development (E13 in the rat, Lauder, 1990; and E11 in the mouse, Pfaar et al., 2002) and acts a neurotrophic factor in early embryonic CNS development and thus even before synapse formation of cortical neurons is completed. Therefore, it aids to establish CNS organization, supporting as well serotonergic (autoregulation) as also non-serotonergic circuit formation during pre- and early postnatal periods (Sodhi and Sanders-Bush, 2004; Vitalis et al., 2007; Daubert and Condron, 2010). 5-HT signaling is involved in cell division, differentiation, survival, and neuronal migration (Dooley et al., 1997; Lavdas et al., 1997; Azmitia, 2001; Vitalis et al., 2007). It further regulates dendrite formation (Vitalis et al., 2007) and synaptogenesis of cortical neurons (Chubakov et al., 1986; Matsukawa et al., 2003) and is released from sprouting axons even before initial synapse formation (Vitalis and Parnavelas, 2003). Genetic or pharmacological disruption of 5-HT signaling leads to disruption of circuit formation as well as alteration of cell morphology, for example in the somatosensory cortex (Gaspar et al., 2003) and interneuronal circuits (Vitalis et al., 2007). Further, disruption of the 5-HT system during early development by stress or drug exposure is associated with altered cognitive ability, neurodevelopmental disorders such as autism spectrum disorders (ASD) and increased incidence of psychopathologies as schizophrenia (Whitaker-Azmitia, 2001).

The myriad of functions of 5-HT in developmental processes corresponds to the expression of a vast amount of receptors, each with its spatial and temporal expression patterns. Seven receptor families for 5-HT have been identified, including the G protein-coupled receptors 5-HT_1_, 5-HT_2_, and 5-HT_4__-__7_ and the only ligand-gated ion channel 5-HT_3_. Thus far, 5-HT_1_ and 5-HT_2_ receptors have received the most attention as effectors of the actions of 5-HT during CNS development (Borella et al., 1997; Azmitia, 2001; Whitaker-Azmitia, 2001; Gaspar et al., 2003; Puig et al., 2004; Bonnin et al., 2006). However, recent evidence suggests that the 5-HT_3_ receptor is involved in several mechanisms which determine the formation of neuronal circuits from embryonic stages onward. In this review, we summarize those recent findings which suggest that 5-HT_3_ receptors emerge as a novel target during the development of the CNS.

## EXPRESSION OF 5-HT_3_ RECEPTORS DURING DEVELOPMENT

The 5-HT_3_ receptor belongs, together with the nicotinergic acetylcholine, the GABA_A_, and the glycine receptor, to the Cys-loop family of ligand-gated ion channels (Barnes and Sharp, 1999; Chameau and van Hooft, 2006; Walstab et al., 2010; Lummis, 2012). To date, two subunits (5-HT_3A_ and 5-HT_3B_) have been identified in rodents (Maricq et al., 1991; Davies et al., 1999), and additional three subunits (3_C_–3_E_) have been identified in humans (Niesler et al., 2007). Functional 5-HT_3_ receptors can be built from the same (only 5-HT_3A_) or different subunits (5-HT_3A_ and 5-HT_3B_ receptor subunits). The receptor composition is crucial for its function (Chameau and van Hooft, 2006; Thompson and Lummis, 2007), in such a way that incorporation of 5-HT_3B_ leads to an increase in single channel conductance and decrease in Ca^2^^+^ permeability (Davies et al., 1999; Noam et al., 2008). Whether the 5-HT_3B_ subunit is a major determinant of 5-HT_3_ receptor function in the CNS is still a subject of debate (van Hooft and Yakel, 2003; Chameau and van Hooft, 2006; Jensen et al., 2008) and appears to, at least in part, depend on species-specific expression patterns. Yet, the putative expression of 5-HT_3B_ subunits as part of a heteromeric 5-HT_3_ receptor complex in the CNS remains of interest, especially in view of the profound effects on Ca^2^^+^ permeability and associated downstream effectors. Most studies of 5-HT_3_ receptor expression and function in the CNS in rodents focus on 5-HT_3A_ receptors and the terms 5-HT_3_ and 5-HT_3A_ are used as equivalent here.

## 5-HT_3_ RECEPTORS ARE EXPRESSED IN CAUDAL EMINENCE-DERIVED IMMATURE AND MATURE INTERNEURONS DURING CORTICOGENESIS

In the CNS, the 5-HT_3_ receptor is first observed in the subpallial ganglionic eminence (GE), the major source of interneurons in the basal telencephalon, at E12.5 (Johnson and Heinemann, 1995; Miquel et al., 1995; Tecott et al., 1995). The rodent GE generates later neocortical GABAergic interneurons which migrate tangentially into the cortical plate. In contrast, neocortical glutamatergic neurons originate in the pallial ventricular zone (VZ) and migrate radially into the cortex (Corbin et al., 2001; Nadarajah and Parnavelas, 2002). Different areas of the GE give rise to various subpopulations of GABAergic interneurons which can be subclassified by their morphology and neuropeptide expression (Flames and Marín, 2005; Rudy et al., 2011; Vitalis and Rossier, 2011).

5-HT_3_ receptor-positive interneurons compromise ~30% of the superficial GABAergic interneurons in the somatosensory cortex (Lee et al., 2010). They coexpress cholecystokinin (CCK), vasoactive intestinal peptide (VIP), and/or neuropeptide Y (NPY) and, at smaller fractions, calretinin (CR) and/or reelin, but not parvalbumin (PV) or somatostatin (SST; Morales and Bloom, 1997; Férézou et al., 2002; Inta et al., 2008; Lee et al., 2010; Vucurovic et al., 2010). Further expressing several morphological and electrophysiological properties, 5-HT_3_ receptor-positive interneurons form a rather heterogeneous group of cells, whose potential common properties remain to be fully characterized (for a recent review, see Rudy et al., 2011). 5-HT_3_ receptor-expressing neocortical interneurons are not only excited by 5-HT but also acetylcholine via nicotinic receptors (Lee et al., 2010). At least a subset of 5-HT_3_ receptor-positive cells receives monosynaptic thalamocortical input leading to strong depolarization of these cells (Lee et al., 2010). Therefore, 5-HT_3_ receptor-expressing cells might be part of potential feedforward inhibitory thalamocortical networks whose sensitivity is potentially regulated by serotonergic and/or cholinergic input (Lee et al., 2010; Rudy et al., 2011). Further discussion of potential functional significance of 5-HT_3_ receptors on these interneurons was published recently (Rudy et al., 2011).

The major source of 5-HT_3_ receptor-expressing neocortical interneurons is the caudal part of the GE (CGE; Lee et al., 2010; Vucurovic et al., 2010). Based on recent publications, there is no expression of 5-HT_3_ receptor in the medial GE (MGE; Lee et al., 2010; Vucurovic et al., 2010), which is the area PV- and SST-expressing cortical interneurons are derived exclusively from (Miyoshi et al., 2007). Note that embryonic 5-HT_3_ receptor expression was mistakenly described in the MGE in earlier publications (Tecott et al., 1995).

Recently, the generation of enhanced green fluorescent protein (EGFP)-expressing 5-HT_3A_ receptor reporter mice by Inta et al. (2008) and the GENSAT (Gene Expression Nervous System Atlas) project allowed for detailed analysis and fate mapping of 5-HT_3_ receptor-positive cells during embryonic corticogenesis (Lee et al., 2010; Vucurovic et al., 2010). 5-HT_3_ receptor-positive superficial neocortical interneurons were found to be generated in the CGE around E13.5–14.5 (Vucurovic et al., 2010). Similar, Miyoshi et al. (2010) described the genesis of cortical interneurons in the CGE to begin at E12.5 and peak at E16.5. Therefore, CGE-derived interneurons are some of the latest cells to integrate into neocortical layers, which by this time point are already populated by other interneurons including MGE-derived interneurons (Butt et al., 2005; Miyoshi et al., 2007; peak of MGE-derived cortical interneuron genesis at E14.5: Miyoshi et al., 2010). 5-HT_3_ receptor-positive neuroblasts thereby migrate at least partly through the neocortical subventricular zone (SVZ) and intermediate zone (IZ; Tanaka and Nakajima, 2012). Further, unlike MGE-derived interneurons, 5-HT_3_ receptor-expressing interneurons do occupy preferentially superficial cortical layers I–III (Miyoshi et al., 2007; Lee et al., 2010; Vucurovic et al., 2010). Additionally, they migrate into the neocortical layers in an “outside-in” (Vucurovic et al., 2010) rather than the “inside-out” integration manner of PV- and SST-expressing interneurons. Such “outside-in” neurogenesis was previously described as a feature of CR interneurons (Rymar and Sadikot, 2007). Interestingly, in contrast to PV-interneurons, the birthdate of these CR-expressing interneurons does not match that of neighboring projection neurons in the corresponding layer (Yozu et al., 2004; Rymar and Sadikot, 2007). This might be true as well for the 5-HT_3_ receptor-positive interneurons. Therefore, 5-HT_3_ receptor-expressing CGE-derived neocortical interneurons might form a group of cells with very specific, yet unknown, characteristics and might follow different migration- and integration cues than other major groups of interneurons like PV-positive interneurons (Lee et al., 2010; Miyoshi et al., 2010).

In grafting experiments, Vucurovic et al. (2010) found that CGE-derived cells also populated several limbic structures including the bed nucleus, hippocampus, and amygdala. These were derived earlier from the CGE then the neocortical cells, which is in line with earlier genesis of interneurons in these regions (Vucurovic et al., 2010).

Furthermore, next to the CGE, embryonic 5-HT_3_ receptor expression was also observed in cells of the entopeduncular area (AEP) and peroptic area (POA; Lee et al., 2010; Vucurovic et al., 2010). The further development of these cells has not been characterized yet. Cells from the POA might contribute to interneurons in the neocortex (Gelman et al., 2009, 2011) and thus it was proposed that the POA might also give rise to 5-HT_3_ receptor-positive interneurons of the neocortex (Rudy et al., 2011). However, Vucurovic et al. (2010) found no evidence of POA cells migrating into neocortical regions but the cells rather contributed, dependent on their birthdate, to cells of the dentate gyrus (DG), amygdala, endopiriform nucleus, and the claustrum.

## 5-HT_3_ RECEPTORS ARE EXPRESSED IN POSTNATAL IMMATURE NEURONS

5-HT_3_ receptors are expressed in migrating neuroblasts in several migratory streams derived from the SVZ in the early postnatal brain (Inta et al., 2008; Vucurovic et al., 2010). The SVZ, and therefore these neuroblasts, are not derived from the CGE but from the lateral GE (LGE). Migratory streams in the early postnatal rodent brain are part of the ongoing neurogenesis and migration of neurons after birth. These migratory streams include the rostral migratory stream (RMS) populating mainly the olfactory bulb (OB), the dorsal migratory pathway (DMP) above the hippocampus directed toward the occipital cortex, the ventral migratory pathway (VMP) heading toward the striatum and nucleus accumbens, and the external migratory pathway (EMP) aiming toward latero-dorsal brain regions (Inta et al., 2008). Neuroblasts of the RMS do not only migrate into and maturate within the OB but also integrate into the cortex (Le Magueresse et al., 2011). Next to cortical interneurons derived from embryonic interneuron genesis, these neuroblasts maturate into a novel, recently described subclass of CR-positive interneurons with unique firing pattern (“small axonless neurons”) which are uniquely generated in the early postnatal period and mainly integrate into deeper layers of olfactory and orbital cortices (Le Magueresse et al., 2011). Additionally, 5-HT_3_ receptor-positive postnatal SVZ-derived neuroblasts, so-called immature white matter interstitial cells, were recently described to populate the corpus callosum (von Engelhardt et al., 2011).

Of the several postnatal migratory streams harboring 5-HT_3_ receptor-positive neuroblasts, only the RMS persists into adulthood as an area of secondary neurogenesis (Alvarez-Buylla and García-Verdugo, 2002; Abrous et al., 2005) containing 5-HT_3_ receptor-positive neuroblasts (Inta et al., 2008; Chen et al., 2012). Similar to early postnatal RMS neuroblasts, they migrate and integrate into the OB, where they maturate to CR- and VIP-positive but calbindin- (CB) negative interneurons. Interestingly, and in contrast to cortical interneurons derived from the CGE, about one-third and one-tenth of the 5-HT_3_ receptor-expressing interneurons in the OB are PV- and SST-positive, respectively (Chen et al., 2012). Adult SVZ neurogenesis is of particular clinical interest because SVZ-derived neuroblasts can migrate into the cortex upon traumatic events or in neurodegenerative diseases to replace cortical neurons. Indeed, upon stroke in adult mice 5-HT_3_ receptor-positive neuroblasts integrate into the cortex and maturate to CR-positive interneurons (Kreuzberg et al., 2010). However, the majority of these cells loses 5-HT_3_ receptor expression upon maturation (Kreuzberg et al., 2010).

To conclude, 5-HT_3_ receptor-expressing neuroblasts are present in several locations in the early postnatal and adult brain. Nevertheless, both the regulation of migration and maturation of embryonic CGE- and adult SVZ-derived neuroblasts as well as the functional role of 5-HT_3_ receptors during these processes are yet unresolved. Only little is known about downstream signaling upon activation of 5-HT_3_ receptors and subsequent Ca^2^^+^ ionic influx. Investigating a potential function of 5-HT_3_ receptors in regulating neuroblast migration and maturation therefore would be promising. Some recent studies proposed regulation of cytoskeletal remodeling in neurons by 5-HT_3_ receptors. For example, 5-HT_3_ receptor agonists were found to promote neurite elongation of GABAergic cortical interneurons (Vitalis and Parnavelas, 2003). Activation of 5-HT_3_ receptors further promotes dendrite formation in primary thalamic neurons *in vitro* (Persico et al., 2006; note contradictory: Lotto et al., 1999). In growth cones, cohesion spots, and dendrites of hippocampal neurons and in human embryonic kidney (HEK) cells, 5-HT_3_ receptors were found to form clusters with the light chain (LC1) of microtubule-associated protein 1B (MAP1B) and the tubulin cytoskeleton (Sun et al., 2008) and these clusters lead to the formation of F-actin-rich lamellipodia (Emerit et al., 2002). 5-HT_3_ receptors follow the tubulin and F-actin networks for receptor routing and precise tuning at the neuronal membrane surface (Grailhe et al., 2004; Ilegems et al., 2004). Further, LC1 might regulate the receptor function in these cells (Sun et al., 2008). Therefore, 5-HT_3_ receptors and the cytoskeleton are highly interacting, which might not only lead to the specific transport of 5-HT_3_ receptors into synaptic sites and regulation of receptor function, but also 5-HT_3_ receptors might evoke signaling involved in cytoskeletal remodeling. 5-HT_3_ receptor activity in immature and mature interneurons might be crucial for their activity as well as development.

Interestingly, it was recently reported that electrophysiological activity is essential for the postnatal correct migration and axonal and dendritic integration of CGE-derived reelin- and CR-, but not VIP-positive neurons (García et al., 2011). Whereas this activity is glutamate-dependent after P3, the source of activity before P3 is yet unclear. Serotonergic input via 5-HT_3_ receptors might be a candidate source of such perinatal activity.

## CONCLUSION I: 5-HT_3_ RECEPTORS ARE A POTENTIAL CENTRAL PART OF MATURATING INTERNEURONS DURING PRE- AND POSTNATAL CORTICAL DEVELOPMENT

**FIGURE 1:**
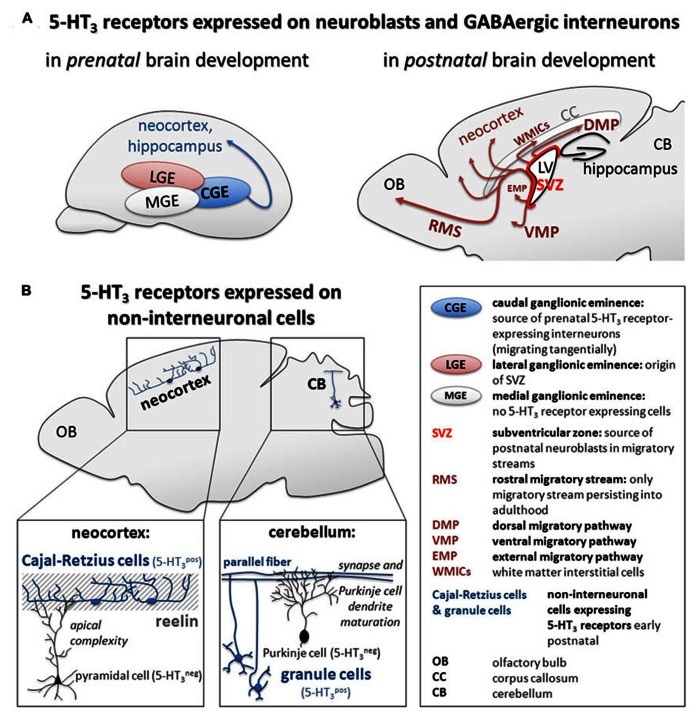
**Summary of (A) 5-HT_**3**_ receptor expression on GABAergic interneurons during pre- and postnatal brain development and **(B)** recently described mechanisms of 5-HT_**3**_ receptor-mediated regulation of maturation of cortical pyramidal cells and cerebellar Purkinje cells in the early postnatal brain**.

5-HT_3_ receptors are expressed on embryonic immature CGE-derived GABAergic interneurons as well as neuroblasts in early postnatal migratory streams and the adult SVZ. Therefore, they might be involved in (fine)regulation of neuronal excitability and thus migration, maturation, and network formation of inhibitory networks from early embryonic to adult stages (**Figure 1**).

## EXPRESSION OF 5-HT_3_ RECEPTORS ON CEREBELLAR GRANULE AND CORTICAL CAJAL–RETZIUS CELLS

Next to the pre- and postnatal central expression of 5-HT_3_ receptors on mature and immature interneurons, recent evidence showed also expression on two specific types of glutamatergic cells: cerebellar granule cells and cortical Cajal–Retzius cells. First, ubiquitous post-/extra- and presynaptic expression of 5-HT_3_ receptors was recently observed in glutamatergic granule cells of the cerebellum within the first three postnatal weeks in rodents (Oostland et al., 2011, 2013). 5-HT_3_ receptors are important for the serotonergic regulation of short-term synaptic plasticity at parallel fiber-Purkinje cell synapses during the early postnatal sensitive period and regulate the maturation state of these synapses (Oostland et al., 2011). They further regulate the time course of early postnatal morphological maturation of Purkinje cells as indicated by higher dendritic length and complexity in 5-HT_3_ receptor knock-out mice and *in vitro* after treatment with a 5-HT_3_ receptor antagonist (Oostland et al., 2013). 5-HT_3_ receptor knock-out animals further show delayed climbing-fiber elimination (Oostland et al., 2013). However, morphology and physiology of Purkinje cells in 5-HT_3_ receptor knock-out mice appears normal in adult mice, thus indicating a narrow postnatal time window of serotonergic, 5-HT_3_ receptor-mediated regulation of cerebellar maturation and connectivity (Oostland et al., 2013). Further research might explore a function of 5-HT_3_ receptors in the development of early life motor coordination and learning.

Second, glutamatergic Cajal–Retzius cells were recently described to express 5-HT_3_ receptors upon birth (Chameau et al., 2009; Lee et al., 2010). Cajal–Retzius cells are transient neurons located in the marginal zones of the neocortex and hippocampus during CNS development (Marín-Padilla, 1998). In the cortex, they are strategically located in layer I, the area where the apical dendrites of pyramidal neurons terminate and secrete the extracellular matrix glycoprotein reelin. Reelin plays a major role as guidance factor for cell migration, cell positioning, and neuronal process outgrowth (Frotscher, 1997). Cajal–Retzius cells in mice are innervated by serotonergic fibers as early as E16. Disruption of the serotonergic system during embryonic development results in lower levels of reelin and a disturbed corticogenesis with disrupted formation of cortical columns (Janusonis et al., 2004). The regulation of corticogenesis by Cajal–Retzius cells is at least partly dependent on 5-HT_3_ receptor signaling (Chameau et al., 2009). Chameau et al. (2009) not only reported expression of 5-HT_3_ receptors specifically on Cajal–Retzius cells (but not on pyramidal neurons), but further established a novel role of 5-HT_3_ receptors, Cajal–Retzius cells, and reelin in the postnatal maturation of cortical pyramidal neurons. Cajal–Retzius cells limit the apical dendritic outgrowth of cortical layer II/III pyramidal cells and thus complexity of cytoarchitecture and network formation. Blocking 5-HT_3_ receptor activity with an antagonist or reelin signaling with an anti-reelin antibody leads to hypercomplexity of the apical dendrites of layer II/III pyramidal neurons in the somatosensory cortex. A similar phenotype is also present in 5-HT_3_ receptor knock-out mice and can be rescued by application of recombinant reelin (Chameau et al., 2009). However, it remains to be investigated if, and how, the release of reelin from Cajal–Retzius cells is directly regulated by 5-HT_3_ receptor activity. Similar findings of possibly indirect regulation of migration and regulation of cytoarchitecture in cortical pyramidal neurons were shown *in vitro* in mixed GABA- and non-GABAergic cortical neuron cultures, where 5-HT_3_ receptor activation inhibited axonal and dendritic outgrowth and dendritic branching only in non-GABAergic cells (Hayashi et al., 2010).

The increased dendritic complexity of cortical layer II/III pyramidal neurons in 5-HT_3_ receptor knock-out mice has been associated with altered cortical spatial organization and connectivity with larger dendritic bundles in layer III tangential sections, whereas spine density was not affected (Smit-Rigter et al., 2011). On a functional level, the increase in dendritic complexity of cortical layer II/III pyramidal neurons in 5-HT_3_ receptor knock-out mice results in a different firing pattern of these cells (van der Velden et al., 2012), suggesting that 5-HT_3_ receptor activity during maturation of neurons is not only important for the wiring of the local microcircuitry, but also consequently for the processing of information within the circuit. As a potential consequence of this disturbed cortical wiring and function, 5-HT_3_ receptor knock-out mice display reduced anxiety-like behavior (Kelley et al., 2003; Bhatnagar et al., 2004) and impaired social behavior (Smit-Rigter et al., 2010), although a direct link between the cortical abnormalities and the behavioral phenotypes remains to be established.

## CONCLUSION II: 5-HT_3_ RECEPTORS REGULATE MATURATION AND DENDRITE COMPLEXITY OF NON-INTERNEURON CELLS

5-HT_3_ receptors regulate the wiring of the local microcircuit in the cortex and the cerebellum by yet unknown either direct or indirect mechanisms via Cajal–Retzius cells and granule cells, respectively. Therefore, 5-HT_3_ receptors may be crucially involved in the formation of higher-level neuronal structures (Figure 1).

## PUTATIVE IMPLICATIONS FOR NEURODEVELOPMENTAL DISORDERS

5-HT_3_ receptors are associated with several psychiatric disorders in humans. Single nucleotide polymorphism, especially the C178T polymorphism in the 5′UTR region of the 5-HT_3_ receptor, were found to be associated with bipolar disorder (Niesler et al., 2001), schizophrenia (Niesler et al., 2001; Thompson et al., 2006), lowered harm avoidance in women (Melke and Westberg, 2003), alcohol and drug dependence (Enoch et al., 2010), lowered activity of amygdala and prefrontal cortex (Iidaka et al., 2005), prefrontal and hippocampal gray matter loss, and early life quality-dependent elevated depressed mood (Gatt et al., 2010a, b). These variants are associated with changes in 5-HT_3_ receptor function and expression (Krzywkowski et al., 2007). However, it has to be noted that 5-HT_3_ receptor genetics is fundamentally different between humans and rodents. 5-HT_3_ receptor expression in humans is much more complicated including additional splice variants of 5-HT_3A_, the possible expression of heteromeric receptors in the CNS, and three additional receptor genes (5-HT_3C__-__E_), whose function and expression in the CNS have yet to be investigated.

The data presented in this review highlights the 5-HT_3_ receptor as a crucial regulator of brain development. This also makes it interesting as novel candidate to be involved brain development pathologies such as ASD. Indeed, several studies present evidence that ASD might be caused by disruptions of the serotonergic system during brain development. Common ASD animal models are based on alterations of prenatal 5-HT levels (Whitaker-Azmitia, 2005; Boylan et al., 2007; Hohmann et al., 2007). Likewise, clinical data from ASD patients points toward a causal relationship of distortion of the serotonergic system and ASD pathology (Anderson et al., 1987; Naffah-Mazzacoratti et al., 1993; Chugani, 2002).

Investigating a potential role of 5-HT_3_ receptors in the development of ASD, it is apparent that 5-HT_3_ receptor knock-out mice display some features similar to ASD symptoms including impaired social behavior (Smit-Rigter et al., 2010) and a reduction in basal anxiety-related behavior (Kelley et al., 2003; Bhatnagar et al., 2004; Smit-Rigter et al., 2010). Further, in line with the potential role of the 5-HT_3_ receptor outlined earlier in this review, these animals display some alterations in neocortical development as hypercomplexity of apical dendrites of cortical layer II/III pyramidal neurons (Chameau et al., 2009) and increased apical dendrite bundling (Smit-Rigter et al., 2011). Disruptions of neocortical development, especially in the balance between excitatory and inhibitory circuits, might at least partially underlie autism neurobiology (Polleux and Lauder, 2004; Levitt, 2005). For example, in parallel with 5-HT_3_ receptor knock-out animals, ASD patients display a cortical column pathology with changes in cortical minicolumn size, number and cellular distribution, and increased cortical volume (Bailey et al., 1998; Casanova et al., 2002; Carper and Courchesne, 2005). Further, reelin signaling was proposed to be impaired in ASD neurobiology (Fatemi et al., 2005). Indeed, 5-HT_3_ gene polymorphisms were recently found to be associated with ASD (Anderson et al., 2009; Rehnström et al., 2009). However, there is yet no evidence of a role of 5-HT_3_ receptors in the neurobiology of ASD.

Finally, recent literature draws attention to the potential risk of disturbing serotonergic circuits during fetal brain development via exposure of fetuses to selective serotonin reuptake inhibitors (SSRIs). The use of SSRIs by pregnant women, especially during the first trimester, may increase the risk of ASD in the offspring (Croen et al., 2011). In mice, early postnatal exposure to SSRIs leads to increased anxiety-like behavior (Ansorge et al., 2004). In addition, *in utero* exposure to fluoxetine leads to life-long abnormalities of cortical cytoarchitecture and increased anxiety-like behavior (Smit-Rigter et al., 2012). These effects were not present in 5-HT_3_ receptor knock-out mice suggesting that the adverse effect of fluoxetine-exposure during brain development might be 5-HT_3_ receptor-dependent (Smit-Rigter et al., 2012).

We conclude that, although current data is still limited, 5-HT_3_ receptors are important for proper brain development. The 5-HT_3_ receptor knock-out mouse has proven to be a valuable tool to elucidate some of the roles of 5-HT_3_ receptors in neuronal development. However, the availability of more advanced tools to knock-out or -down 5-HT_3_ receptors in a more spatially and temporally controlled manner is eagerly anticipated.

## Conflict of Interest Statement

The authors declare that the research was conducted in the absence of any commercial or financial relationships that could be construed as a potential conflict of interest.
